# ADHD Symptoms in Childhood and Big Five Personality Traits in Adolescence: A Five-Year Longitudinal Study in Girls

**DOI:** 10.1007/s10802-024-01204-x

**Published:** 2024-05-29

**Authors:** Laura J. Bell, Oliver P. John, Stephen P. Hinshaw

**Affiliations:** 1 Department of Psychology, University of California, Berkeley, USA; 2 Department of Psychiatry and Behavioral Sciences, University of California, San Francisco, USA

**Keywords:** ADHD, Big Five, Personality development, Longitudinal, Childhood, Adolescence

## Abstract

Attention-deficit/hyperactivity disorder (ADHD) is a childhood-onset neurodevelopmental condition characterized by developmentally extreme and impairing symptoms of inattention and/or hyperactivity/impulsivity. Great interest has emerged in the ways ADHD and its underlying symptom dimensions relate to the development of personality traits. Much extant research on this topic is cross-sectional, relying on self-report measures and male samples. Herein, we present data from a prospective, longitudinal study of a socioeconomically and racially diverse sample of girls, including those with ADHD and a matched neurotypical comparison sample. We examined how parent- and teacher-reported ADHD in middle childhood relate to self-reported Big Five personality traits in adolescence. As expected, childhood ADHD diagnosis prospectively predicted lower self-reported Conscientiousness, lower Agreeableness, and higher Neuroticism in adolescence. With ADHD diagnosis covaried, Inattention (IA) predicted only low Conscientiousness, Hyperactivity/Impulsivity (HI) predicted only low Agreeableness, and neither predicted adolescent Neuroticism. An exploratory moderator analysis showed that family income moderated the effects of IA and HI on the negativity of adolescent self-descriptions of their own personalities, with more pronounced negative effects for girls in families with higher (rather than lower) income. Familial pressures to achieve in higher-income families may be linked to more pronounced negative ramifications of ADHD on personality development.

**Supplementary Information:**

The online version contains supplementary material available at 10.1007/s10802-024-01204-x.

## Introduction

Attention-deficit/hyperactivity disorder (ADHD), a neurodevelopmental condition characterized by pervasive and impairing patterns of attentional dysregulation, hyperactivity, and/or impulsivity, shows substantial overlap with several dimensions of both personality and temperament. All three constructs (ADHD, temperament, and personality) tap aspects of self-regulation, including behavioral and emotional control. Insufficient literature assesses the interplay among these constructs across development. Through the present prospective, longitudinal investigation, we attempt to bridge this gap in knowledge by examining the linkages between childhood ADHD symptoms and adolescent personality dimensions. In so doing, we pay careful attention to methodologic issues that might spuriously inflate such linkages.

ADHD is characterized by two symptom dimensions—inattention (IA) and hyperactivity/ impulsivity (HI)—either (or both) of which may be present in diagnosed ADHD. Inattentive symptoms include behaviors such as making frequent careless mistakes, having difficulty paying attention, getting distracted easily, disorganization, and forgetfulness, whereas hyperactive-impulsive symptoms include frequent fidgeting, climbing or running at inappropriate times, difficulty working or playing quietly, and frequently interrupting (American Psychiatric Association, 2022). Like the categorical ADHD diagnosis, the two symptom dimensions of IA and HI are substantially heritable (Faraone & Larsson, 2019), usually emerge in childhood, and often become evident in the context of schooling.

In contrast, personality traits refer to a person’s habitual patterns of thinking, feeling, and behaving (John, 2021). They are somewhat less heritable^1^ than ADHD symptom dimensions and appear to develop out of transactions between youth and their environments from early childhood through adolescence and even early adulthood (Roberts et al., 2006). Thus, personality traits may be more susceptible to environmental influence (Srivastava et al., 2003)—and potentially modifiable—than ADHD per se or its symptom dimensions, especially during early and middle adolescence (Soto et al., 2011).

Here we focus on the personality traits defined by the Big Five taxonomy, which posits five fundamental, bipolar factors: (a) Conscientiousness, encompassing traits such as being responsible, organized, and persistent; (b) Agreeableness, including compassion, respect, and trust; (c) Neuroticism, referring to tendencies toward anxiety, depression, and mood volatility; (d) Openness to Experience, encompassing curiosity and a tendency for aesthetic appreciation; and (e) Extraversion, involving being outgoing and assertive (John et al., 2008; McCrae & Costa, 1999; Soto & John, 2017). Prior research suggests that three of the Big Five personality domains—specifically Conscientiousness, Agreeableness, and Neuroticism—are consistently linked to ADHD and its symptom dimensions. In contrast, examinations of Openness to Experience and Extraversion do not reveal consistent associations, with some variability depending on participant age, sample type (community versus clinical), other participant characteristics, and methodology (Gomez & Corr, 2014).

Although research on personality and developmental psychopathology has often proceeded independently, previous cross-sectional research suggests substantial overlap between measures of personality and ADHD (De Pauw & Mervielde, 2010; Gomez & Corr, 2014). For example, some items on the Big Five Inventory (BFI; John & Srivastava, 1999) assess aspects of task focus and distractibility that are similar to the IA symptoms listed in the *Diagnostic and Statistical Manual of Mental Disorders* (DSM; American Psychiatric Association, 2022). A core question is whether childhood ADHD symptom dimensions are related to adolescent personality constructs, especially when similar items from measures of either domain are removed and when informants differ across age.

### Personality and ADHD: Prior Research

Numerous studies have shown that ADHD—especially IA symptoms—is substantially negatively correlated with the Big Five dimension of Conscientiousness, with correlation sizes estimated to be at least medium-to-large^2^ (*r* = -0.43 for ADHD, *r* = -0.52 for IA, and *r* = -0.40 for HI; see meta-analytic review by Gomez & Corr, 2014). In addition, ADHD—especially HI symptoms—is associated with various interpersonal difficulties, including interrupting others, higher levels of peer conflict, and aggression (Gardner & Gerdes, 2015; Hinshaw, 2018). Correspondingly, several studies have shown that ADHD (i.e., HI) negatively correlates with Agreeableness in the Big Five taxonomy, with correlations estimated to range from medium-to-large (*r* = -0.31 for ADHD, *r* = -0.22 for IA, and *r* = -0.30 for HI; Gomez & Corr, 2014).

Moreover, although emotional dysregulation (often manifesting as “emotional impulsivity”) is not part of the DSM diagnosis of ADHD, it is highly linked to the ADHD syndrome (see Barkley, 2015; Faraone et al., 2019). Regarding the Big Five, this association has been demonstrated in the positive correlations between Neuroticism and ADHD—including its two symptom dimensions, IA and HI, with associations ranging from 0.39 for ADHD, 0.35 for IA, and 0.18 for HI (thus, in the medium to large range; see Gomez & Corr, 2014). Finally, Gomez and Corr’s (2014) meta-analysis suggests no significant associations between ADHD, IA, or HI and Openness to Experience nor between ADHD, IA, or HI and Extraversion.

The meta-analysis by Gomez and Corr (2014) spans clinical, community, adult, and child samples, with the strong suggestion that associations between ADHD symptoms and personality traits (i.e., Conscientiousness, Agreeableness, and Neuroticism) are present across different samples and age groups. In general, the correlations between ADHD and these three Big Five dimensions tend to be somewhat larger in (a) clinical samples than in community samples and (b) in child and adolescent samples than in adult samples. In their conclusions, Gomez and Corr (2014) emphasize one important limitation of their meta-analysis: Most of the research available for their review used cross-sectional designs and adult participants. Even though the link between ADHD and personality is an inherently developmental issue, few studies have examined these links longitudinally.

A notable exception is the investigation of Miller et al. (2008), who examined prospective links between a childhood ADHD diagnosis and self-rated personality in adolescence. Here, personality was measured using the revised NEO Personality Inventory (NEO PI-R; Costa & McCrae, 1992) and childhood ADHD diagnostic status was assessed via: (1) a screener using teacher-ratings on the IOWA Conners (Loney & Milich, 1982) inattention/overactivity scale, followed by (2) a parent-reported Diagnostic Interview for Children (DISC; Shaffer et al., 1989) and Child Behavior Checklist (CBCL; Achenbach, 1991) for children who crossed the clinical cutoff on the teacher measure, and (3) clinical classification using DSM-III-R or DSM-IV ADHD criteria. Key findings were that childhood ADHD (categorically defined) was negatively predictive of adolescent Conscientiousness, regardless of the persistence of ADHD into adolescence. On the other hand, childhood ADHD negatively predicted Agreeableness and positively predicted Neuroticism.

Crucially, however, Miller et al. (2008) examined ADHD solely as a dichotomous variable and did not examine ADHD symptom dimensions separately. Additionally, the entirety of the sample had childhood diagnoses of ADHD Combined Type, limiting generalizability to youth with predominantly inattentive symptoms. Finally, parallel to much research on ADHD and personality, the longitudinal sample in this study consisted primarily of boys (88%), with only 20 girls. Thus, questions remain about the generalizability of such findings to girls with ADHD.

This gap is particularly important because females tend to have higher rates of exclusively inattentive symptoms than boys (Mowlem et al., 2019), along with lower rates of externalizing comorbidities (Hinshaw et al., 2022). Note that mean-level sex differences in personality are present as well: Females tend to score higher on Agreeableness, which generally associates with more adaptive development—but also higher on Neuroticism, which could be a risk factor for mental health concerns (McCrae & Costa, 1999). Overall, to better understand ADHD-personality linkages, especially given the longstanding neglect of females in the literature on ADHD, there is a clear need to focus on girls, to deploy longitudinal designs, and to assess not only ADHD diagnostic status but also the two symptom dimensions of IA and HI in the same investigation.

In addition, measurement issues require careful consideration, especially shared method variance, which can artifactually elevate associations between two variables of interest (Campbell & Fiske, 1959). Of foremost importance are (a) using different data sources for the measurement of ADHD and the measurement of personality (i.e., not using self-reports for both) and (b) eliminating any direct item overlap between symptom dimensions and personality scales (e.g., Inattention and Conscientiousness).

Finally, investigators should consider the family backgrounds in which children with or without ADHD are developing, as these contexts have potential implications for personality development. The bulk of personality research in this area has focused on college-student samples, which tend to overrepresent young adults from higher-income families who grew up in what Luthar (2003) and Korous et al. (2023) have described as a “culture of affluence”—a context placing pressure on children and adolescents to excel academically. It is important to study ADHD-personality links in samples that are representative of low-income as well as middle- and upper-middle-class families. Through exploratory yet empirically guided examination, we probe whether links between ADHD symptoms in childhood and personality in adolescence might differ in strength as a function of family income—in particular, whether these links are actually weaker in lower-income families (compared to higher-income families, in which pressures for achievement are likely to be quite salient). Specifically, we examine whether children who have childhood ADHD symptoms and grow up in higher-income families feel worse about themselves in adolescence than those from lower-income families.

### The Present Research Questions

The current investigation was designed to examine the prospective link between childhood IA and HI (as well as the diagnostic category of ADHD per se) and adolescent personality traits, using different data sources at different time points to minimize shared method variance. Our sample provides a unique opportunity for such examination in females, who are typically understudied in the area of ADHD (Hinshaw et al., 2022). Based on existing literature, we hypothesize that childhood IA, HI, and categorical ADHD diagnosis will all (a) negatively predict adolescent Conscientiousness and Agreeableness and (b) positively predict adolescent Neuroticism. Given the lack of cross-sectional correlations between ADHD and either Openness to Experience or Extraversion (Gomez & Corr, 2014), we anticipate no significant longitudinal predictions to these two Big Five dimensions.

More specifically, we expect that only IA (and not HI) symptoms will predict adolescent Conscientiousness when ADHD diagnosis is statistically taken into account. Conversely, we also predict that only HI (and not IA) symptoms will predict Agreeableness beyond the effects of ADHD diagnosis. Any prospective effects related to Neuroticism are of real theoretical interest, because emotional instability or dysregulation is not part of the definition and measurement of ADHD, raising the possibility that higher levels of Neuroticism (anxiety, depression, negative self-concept) in adolescence may be a consequence of growing up with ADHD rather than serving as a preexisting disposition in childhood. Finally, regarding family financial standing, we examined whether a “culture of affluence” (Luthar, 2003) may amplify these kinds of effects. In other words, we suspect that linkages between childhood ADHD and negative self-views in adolescence may be stronger for families of higher socioeconomic status. Finally, we assiduously removed any directly overlapping items from our ADHD and personality measures, as failing to do so could spuriously inflate predictive associations.

## Method

### Overview of Procedures

In this research we use data from the Berkeley Girls with ADHD Longitudinal Study (BGALS), a longitudinal dataset that assessed ADHD in childhood and Big Five personality traits in adolescence (for an overview, see Owens et al., 2016). We focus on data collected during childhood (Wave 1), *M* = 9.6 years, *SD* = 1.68 (range 6–12 years), and during adolescence (Wave 2), *M* = 14.2 years, *SD* = 1.68 (range 11–18 years; 97% were between 11 and 16 years), when participants were old enough to rate their own personality traits. The retention rate between Wave 1 and Wave 2 was 92%.

The BGALS project was approved by the Committee for Protection of Human Subjects (i.e., the Institutional Review Board) at the University of California, Berkeley. Written consent and assent were obtained from both legal guardians and youth participants, respectively, at each wave.

### Participants

Participants were 228 girls, either with (*N* = 140) or without (*N* = 88) carefully diagnosed childhood ADHD. They were recruited from schools, doctors’ offices, mental health care settings, and advertisements. For the ADHD subsample, 93 girls met criteria for ADHD-Combined type (i.e., high levels of both IA and HI) and 47 for ADHD-Inattentive type (high levels of IA only). The sample was racially/ethnically and socioeconomically diverse, with representation of families with working class jobs (e.g., truck driver, custodian) and families receiving public assistance, along with upper-middle class families with higher incomes. The clinical (ADHD) and comparison samples were group-matched on age, neighborhood, and ethnicity. They participated alongside one another in enrichment summer research programs in 1997, 1998, or 1999 (see Hinshaw, 2002, for details on Wave 1 sample ascertainment and baseline findings). The sample was 53.9% Caucasian, 25.5% African-American, 11.8% Latinx, 8.3% Asian American, and 0.5% Native American. Median yearly gross household income at the baseline assessment in the late 1990s was $60,000–70,000.

In adolescence (Wave 2), 24 participants from Wave 1 did not complete the Big Five measure. To test whether these participants significantly differed from the longitudinal participants with respect to baseline measures, we conducted t- and Mann–Whitney U tests. As expected, participants with more severe ADHD symptoms were less likely to complete the adolescent personality measures. This loss of participants with more pronounced ADHD symptoms is likely to reduce, rather than enhance, any effect sizes reported here, so we believe it is more conservative to use the existing data rather than to impute missing data points (particularly because there is no other personality measure in the study). The final sample of 204 participants included 40 ADHD-Inattentive, 82 ADHD-Combined, and 82 comparison girls.

In terms of power, we offer two considerations. On the one hand, when this longitudinal study was initiated, it was considered unusually large, and it remains today the largest longitudinal study of girls with ADHD data in childhood and personality data in adolescence. Given that ADHD and personality were measured with different data sources as well as five years apart, most of the usual measurement errors (same time of measurement, same data sources) have been ruled out; stronger measurement reduces error in statistical analyses and increases our trust in the findings. On the other hand, from today’s statistical perspective, we wish the sample were larger than the 204 participants we have. Nonetheless, on the basis of our literature review, we expected at least small-to-medium sized correlations and betas (i.e., above 0.20). With a sample of *N* = 200, we have considerable power to detect these kinds of effects according to G*Power, with power of 0.80 and above for population *r*s and *betas of* 0.25 with 2 predictors. Testing the interactions with one predictor and one moderator variable is more challenging with this sample size but is aided, again, by stronger measurement, by lack of correlations between predictor and moderator, and by our setting a stricter alpha level of *p* < 0.01.

### Inattention (IA) and Hyperactive/Impulsive (HI) Symptoms in Childhood (Wave 1)

A growing literature supports a bifactor model of ADHD (incorporating both the overarching ADHD diagnosis/syndrome and its two subordinate symptom dimensions of IA and HI; see Arias et al., 2018). At Wave 1, measures of childhood IA and HI were collected via validated parent and teacher reports on the Swanson, Nolan, and Pelham (SNAP-IV) questionnaire, a dimensionalized checklist of the *DSM-IV (*American Psychiatric Association, 1994) items for ADHD symptom dimensions (IA, HI), and oppositional defiant disorder (ODD, not included in the present study). In response to the prompt, “Have you noticed that this child…”, informants rated each item on a 4-point Likert metric: *0* = *Not at all, 1* = *Just a little, 2* = *Pretty much,* and *3* = *Very much*. Example items include: “Is easily distracted by extraneous stimuli,” “Has difficulty organizing tasks and activities,” “Fidgets with hands or feet or squirms in seat,” and “Blurts out answers to questions before the questions have been completed.”

The SNAP-IV is widely used as an ADHD screener (e.g., in the Multimodal Treatment Study of Children with ADHD [MTA]; MTA Cooperative Group, 1999) and has good to excellent internal consistency (0.71–0.97), test–retest reliability, and validity statistics (Bussing et al., 2008; Solanto & Alvir, 2009; Swanson, 1992). Inter-rater reliability between parent- and teacher-report has been estimated to be in the range of *r* at or above 0.4 (Bussing et al., 2008), as is common among measures of attention and disruptive behavior problems (see Swanson et al., 1999, for detailed discussion of parent-teacher discrepancies on childhood rating scales).

For each participant, composite scores were calculated for each reporter on each symptom dimension by calculating the mean rating of the corresponding items. A cross-observer aggregate score was then calculated for each symptom dimension by averaging the composite scores of the primary caregiver and teacher. Descriptive statistics for each aggregate score at Wave 1 are as follows: IA (*M* = 1.45, *SD* = 0.97) and HI (*M* = 1.00, *SD* = 0.84).

The primary caregiver was the participant’s mother or other female relative for all but two participants, for whom their fathers were considered the primary caregivers. One participant was missing a teacher report but was homeschooled, so the primary caregiver’s mean ratings were used in place of the cross-observer aggregate scores. For observer reports (primary caregiver and teacher) and the cross-observer aggregates, internal consistency (*α*) of IA and HI scales ranged between 0.94 and 0.98. Primary caregivers and teachers agreed substantially on both Wave 1 IA (*r* = 0.79) and HI (*r* = 0.66). All analyses reported in subsequent text and tables refer to the multi-informant aggregate scores for IA and HI.

### ADHD Diagnostic Status in Childhood (Wave 1)

*DSM-IV* diagnostic status for childhood ADHD (present/absent) was determined using the Diagnostic Interview Schedule for Children (4th ed.; DISC-IV; Shaffer et al., 2000) via parent-report. That is, following a given child’s surpassing of symptom-based thresholds on the SNAP-IV, a DISC-IV structured interview occurred with the family. The child had to meet full criteria for either the Inattentive or Combined subtype of ADHD on the DISC-IV for study inclusion. Study clinicians supplemented the DISC-IV interview with the use of no more than two items on the teacher-report form of the SNAP-IV; as in the MTA study (see Hinshaw et al., 1997), items on the teacher-report SNAP-IV with ratings of 2 or 3 on the 0–3 Likert scale were considered positive symptom endorsements. The comparison sample did not meet criteria for ADHD. For details, see Hinshaw (2002).

The DISC-IV parent-report has acceptable to excellent test–retest reliability and testing validity (Shaffer et al., 2000). The DISC-IV parent-report interview’s 1-year test–retest reliability has been estimated to be approximately 0.79 for the categorical ADHD diagnosis among clinical samples (Shaffer et al., 2000). In a community sample, an earlier version of the parent-report DISC’s ADHD section had test–retest reliability estimates of 0.60 for the categorical ADHD diagnosis, 0.84 for symptom counts, and 0.77 for the criterion counts (Shaffer et al., 1996, 2000).

### ADHD Diagnostic Status and Symptom Dimensions in Adolescence (Wave 2)

The same measures were obtained again five years later when the participants were, on average, 14.2 years old.

### Personality in Adolescence: Big Five Inventory (Wave 2)

Personality characteristics in adolescence were measured using self-report on the 44-item Big Five Inventory (BFI; John & Srivastava, 1999; John et al., 1991), a frequently used and well-studied measure that generates reliable and valid composite scores for each Big Five dimension. Among community samples of college students and adults, alpha reliabilities for the five short scales are substantial, averaging 0.80 and above (e.g., John & Srivastava, 1999; Nigg et al., 2002), and convergent correlations with the other two commonly used Big Five measures were impressive, averaging 0.80 with Goldberg’s Trait Descriptive Adjectives (TDA) and 0.77 with Costa and McCrae’s NEO Five Factor Inventory (NEO-FFI); in contrast, the average convergent validity correlation between the TDA and NEO-FFI was only 0.68 (see John et al. 2008, Table 4.5). The 44-item BFI has also been shown to predict peer ratings of personality and a wide range of affective, behavioral, and cognitive outcome variables, such as academic performance (e.g., DeYoung 2006).

The average reading level for the BFI is the fifth grade (Benet-Martinez & John, 1998), and the five-factor structure of this measure holds for youth as young as 11 years of age (see Soto et al., 2008). At age 14 (the mean age in our sample), the average factor congruence with the adult factor structure averaged 0.99, indicating that the BFI functioned as well in this age group as it does in adults. Previous research has examined personality development across adolescence (e.g., Soto et al., 2011); age differences are generally small in size (*r*s below 0.20), with Conscientiousness and Agreeableness decreasing from age 11 to 15 for both boys and girls, and Neuroticism increasing from 11 to 15 in girls (Soto et al., 2011).

Nonetheless, we used a version of the BFI adapted for adolescents, which includes simplified versions of items difficult to understand for younger age groups, based on previous research (Soto et al., 2008) and an expert panel. For example, the Conscientiousness item “Does a thorough job” was changed to “Does things carefully and completely” because “thorough” is a more difficult word and because “job” may be understood as referring to a formal work context not yet applicable to young adolescents.

The BFI is administered using the prompt, “I see myself as someone who…,” followed by the 44 BFI items. Each adolescent girl rated herself on a 5-point Likert scale with the following response options: *1* = *Disagree strongly*, *2* = *Disagree a little*, *3* = *Neither agree nor disagree*, *4* = *Agree a little*, and *5* = *Agree strongly*. Sample items are “Does things carefully and completely” for Conscientiousness, “Is kind and considerate to almost everyone” for Agreeableness, and “Worries a lot” for high Neuroticism. We generated composite scores for each of the Big Five traits by taking the mean rating of the items for each Big Five dimension.

Descriptive statistics and alpha reliabilities for each self-reported personality dimension in Wave 2 were consistent with previous studies, and the alpha reliability of the five scales in this sample was substantial, averaging 0.78: Conscientiousness (*M* = 3.39, *SD* = 0.71, α = 0.81), Agreeableness (*M* = 3.86, *SD* = 0.62, α = 0.79), Neuroticism (*M* = 2.64, *SD* = 0.68, α = 0.77), Openness to Experience (*M* = 4.07, *SD* = 0.54, α = 0.75), and Extraversion (*M* = 3.71, *SD* = 0.67, α = 0.76). See Appendix A, Table S1 for descriptive statistics.

### Negative Self-Views in Adolescence (Wave 2)

To measure negative self-views, we computed a higher-order factor of the Big Five identified initially by Digman (1997) and replicated by Paulhus and John (1998) and DeYoung (2006). This factor encompasses three of the Big Five dimensions—Conscientiousness, Agreeableness, and Emotional Stability (i.e., reverse-coded Neuroticism). Digman (1997) viewed this factor “as a broad collection of traits that actually are socially desirable” (p. 1249). Here we used the adolescent self-reports on this superordinate factor, reverse-keyed in the undesirable direction, to index generally negative self-views. For example, adolescents scoring high on this index described themselves as lazy (low Conscientiousness), rude to others (low Agreeableness), and moody (high Neuroticism), reflecting substantially negative self-perceptions. This negative self-views index was computed by averaging the three BFI scales (i.e., reverse-coded Conscientiousness, reverse-coded Agreeableness, and Neuroticism). In the present sample, the alpha reliability of this three-scale index of negative self-views was 0.71.

### Family Income

As suggested by Luthar (2003), we used annual total gross family income to measure this key indicator of socioeconomic status. Family income information was collected via parent report at baseline (Wave 1). Parents reported their annual total gross family income in the following categories: 1 = < $10,000, 2 = $10–20,000, 3 = $20–30,000, 4 = $30–40,000, 5 = $40–50,000, 6 = $50–60,000, 7 = $60–70,000, 8 = $70–75,000, 9 = > $75,000. Descriptive statistics for the sample with complete W2 BFI and income data were as follows: *N* = 200, *M*_*income*_ = 6.55, *SD*_*income*_ = 2.53. After adjusting for Consumer Price Index inflation since 1997, the categories can be approximated as the following in today’s dollars: 1 = < $19,385, 2 = $19,385 to $38,770, 3 = $38,770 to $58,155, 4 = $58,155 to $77,540, 5 = $77,540 to $96,925, 6 = $96,925 to $116,310, 7 = $116,310 to $135,695, 8 = $135,695 to $145,388, 9 = > $145,388 (U.S. Bureau of Labor Statistics, n.d.).

### Addressing Item Overlap on Key Measures

To address the potential for artifactual overlap between SNAP-IV symptom scales and BFI scales, we created a list of all items, identifying three that overlapped almost entirely across the two instruments. Then, using a random number generator, we chose one item from each pair of overlapping items and removed it from the scale (see Appendix A, Table S2). The aim was to prevent spurious predictions from nearly identical items across ADHD and personality domains. We removed the item “Is easily distracted” from the SNAP-IV IA scale and the items “Can be somewhat careless” and “Tends to be disorganized” from the BFI Conscientiousness scale. We then re-calculated the average rating for each scale with the smaller sets of items, reconducting all analyses both with and without the removed items as a check on robustness. Analyses revealed nearly identical patterns. To be conservative statistically, in all results reported below, we use scales with item overlap removed. It is worth noting that we observed no overlap between the HI items with either Agreeableness or Neuroticism on the BFI.

### Preregistration Statement

The present study was not preregistered because it involved analyses of existing longitudinal data. Although multiple publications have previously used the ADHD diagnosis and symptom data used in the present research with respect to other outcomes of interest, no papers have examined the adolescent personality data, which are the focus of this report.

To reassure the reader that hypotheses were not made post hoc, we note that we derived hypotheses from previous work establishing cross-sectional associations between ADHD (diagnosis, IA, HI) and Conscientiousness, Agreeableness, and Neuroticism (e.g., Gomez & Corr, 2014; Nigg et al., 2002). The hypothesis that family income may moderate these effects is exploratory and based on prior work by Luthar (2003), who argued for these moderation effects in different domains of psychopathology.

## Data Analytic Plan

We first calculated pairwise correlations (Pearson’s *r*) among childhood ADHD diagnostic status (yes/no), ADHD symptom dimensions of IA and HI in childhood and adolescence, and the Big Five dimensions in adolescence.

Second, for the Big Five dimensions of interest here (Conscientiousness, Agreeableness, and Neuroticism), we tested whether childhood IA and HI would predict each personality dimension, even when the effect of ADHD diagnosis was statistically controlled. We used multiple regression analyses in which we entered both the ADHD diagnosis and the symptom dimension as predictors. We addressed whether childhood symptom dimensions would predict adolescent Big Five traits independent of ADHD diagnostic status, given that a growing literature supports a bifactor model of ADHD that incorporates both the overarching ADHD syndrome and its two subordinate symptom dimensions of IA and HI (Arias et al., 2018).

Third, to test our exploratory hypothesis about the potential role of family income, we first examined pairwise correlations between family income and each core variable in our study. Then we conducted two moderated multiple regression analyses in which one of the symptom dimensions (either IA or HI), family income, and the interaction between the symptom dimension and income were all entered as predictors of the negative self-views index. To assist with interpretability, all variables were z-scored prior to these analyses. Interactions are visualized by plotting regression lines for each symptom dimension predicting negative self-views separately for two groups: higher-income families (with incomes 1 SD above the mean) and lower-income families (with incomes 1 SD below the mean).

Finally, we conducted two robustness tests to ensure that the regression results were not related to the influence of either age differences or racial/ethnic differences among the participants. In one set of regression analyses, we covaried age. In the second set, we covaried the three major racial/ethnic groups comprising our sample, namely Caucasian, African American, and Latinx, using dummy coded variables.

## Results

### Correlations

Table 1 shows all pairwise correlations among core variables of interest. ADHD diagnosis in childhood was negatively correlated with Conscientiousness (*r* = -0.33, large effect size) and Agreeableness (*r* = -0.20, medium effect size) in adolescence, positively associated with Neuroticism (*r* = 0.19, small effect size), and not significantly associated with Openness to Experience or Extraversion. As for specific childhood ADHD symptom dimensions, the correlation between IA and Conscientiousness (*r* = -0.37) was large, whereas IA’s correlation with Agreeableness (*r* = -0.19) was small. In contrast, HI’s correlation with Agreeableness (*r* = -0.25) was medium whereas its correlation with Conscientiousness (*r* = -0.19) was small. The correlations with Neuroticism were similar for the two symptom dimensions: *r* = *0.1*6 (small) for IA and *r* = 0.19 (small) for HI; in comparison, the correlation between dichotomous diagnostic status and Neuroticism was 0.19.

**Table 1:** Longitudinal Correlations of ADHD Diagnosis and Observer-reported Symptoms with Self-reported Big Five Personality

Measures			Childhood ADHD Measures						Adolescent Big Five Personality				
			ADHD Diagnosis		IA		HI		girl self-report				
			W1	W2	W1	W2	W1	W2	C	A	N	O	E
ADHD Measures	ADHD Diagnosis	W1	—	0.63***	0.89***	0.68***	0.76***	0.56***	-0.33***	-0.20**	0.19**	-0.03	0.06
		W2		—	0.62***	0.70***	0.56***	0.56***	-0.33***	-0.25***	0.14*	0.03	0.03
	Inattention	W1			—	0.68***	0.75***	0.49***	-0.37***	-0.19**	0.16*	-0.03	0.07
		W2				—	0.57***	0.70***	-0.41***	-0.31***	0.17*	0.02	0.06
	Hyperactivity/ Impulsivity	W1					—	0.67***	-0.19**	-0.25***	0.19**	0.09	0.12
		W2						—	-0.18*	-0.020**	0.16*	0.13	0.13
Adolescent Big Five Personality (girl self-report)	Conscientiousness	W2							—	0.56***	-0.34***	0.24**	0.10
	Agreeableness	W2								—	-0.44***	0.22**	0.10
	Neuroticism	W2									—	-0.02	-0.10
	Openness	W2										—	0.31***
	Extraversion	W2											—

Mean age was 9.5 years at Wave 1 (childhood) and 14.2 years at Wave 2 (adolescence). ADHD Diagnosis is a categorical (yes/no) variable based on a clinician’s evaluation of parental and teacher reports as well as an assessment of onset and functional impairment. Dimensional inattention and hyperactivity/impulsivity scores were obtained with the Swanson, Nolan, & Pelham (SNAP-IV) questionnaire, with reports by the primary caregiver and a teacher combined. As shown in Table S2 in the Appendix, item overlap between inattention and Conscientiousness was removed.

Key hypothesized longitudinal effects from Wave 1 (W1) ADHD to Wave 2 (W2) personality are in bold.

^*^ *p* < 0.05; ^**^ *p* < 0.01; ^***^ *p* < 0.001

### Multiple Regressions: Do ADHD Symptom Dimensions Predict Personality Traits Beyond ADHD Diagnostic Status?

Results of multiple regressions are shown in Table 2. As hypothesized, results revealed negative, longitudinal associations between: (a) childhood IA and adolescent Conscientiousness and (b) childhood HI and adolescent Agreeableness, even when statistically adjusting for ADHD diagnosis. When predicting adolescent Conscientiousness with ADHD diagnosis covaried, IA still had a significant effect, *β* = -0.34. However, HI did not significantly predict Conscientiousness, *β* = 0.14, when covarying ADHD diagnosis. Conversely, when predicting adolescent Agreeableness while covarying ADHD diagnosis, HI still had a significant effect, *β* = -0.24, but IA did not, *β* = -0.07. Finally, when predicting adolescent Neuroticism, the R^2^ for the regression was significant, but neither IA (*β* = -0.04) nor HI (*β* = 0.10) had effects independent of ADHD diagnosis. In other words, ADHD in childhood was related to Neuroticism in adolescence, as shown in Table 1, but neither of the two symptom dimensions had an effect above and beyond categorical ADHD diagnosis.

**Table 2:** Do the ADHD Symptom Dimensions Predict Adolescent Personality Even When ADHD Diagnosis is Controlled? Multiple Regressions Separating ADHD Symptom Dimensions from ADHD Diagnosis

ADHD Measures (Childhood)		Big Five Personality, self-report (Adolescence)					
		Conscientiousness		Agreeableness		Neuroticism	
		β	*R*^*2*^	β	*R*^*2*^	β	*R*^*2*^
Inattention		-0.34^*^	0.13^***^	-0.07	0.04^*^	-0.04	0.04^*^
ADHD Diagnosis		-0.03		-0.13		0.22	
Hyperactivity/Impulsivity		0.14	0.12^***^	-0.24^*^	0.06^**^	0.10	0.04^*^
ADHD Diagnosis		-0.44^***^		-0.01		0.11	

*N* = 204. Mean age was 9.5 years at Wave 1 (childhood) and 14.2 years at Wave 2 (adolescence). ADHD Diagnosis is a categorical (yes/no) variable based on a clinician’s evaluation of parental and teacher reports as well as an assessment of onset and functional impairment. Dimensional inattention and hyperactivity/Impulsivity scores were obtained with the Swanson, Nolan, & Pelham (SNAP-IV) questionnaire, with reports by the primary caregiver and a teacher combined. As shown in Table S2 in the Appendix, item overlap between inattention and Conscientiousness was removed. Key hypothesized effects are in bold.

^*^ *p* < 0.05; ^**^ *p* < 0.01; ^***^ *p* < 0.001

### Moderation Analyses Involving Family Income

Family income was not significantly related to any of the three personality traits; none of the three correlations reached 0.10. For the ADHD symptom dimensions, all correlations with family income were below 0.20 in magnitude, and only the correlation for childhood HI was significant, *r* = -0.16, *p* < 0.05.

**Fig. 1:**
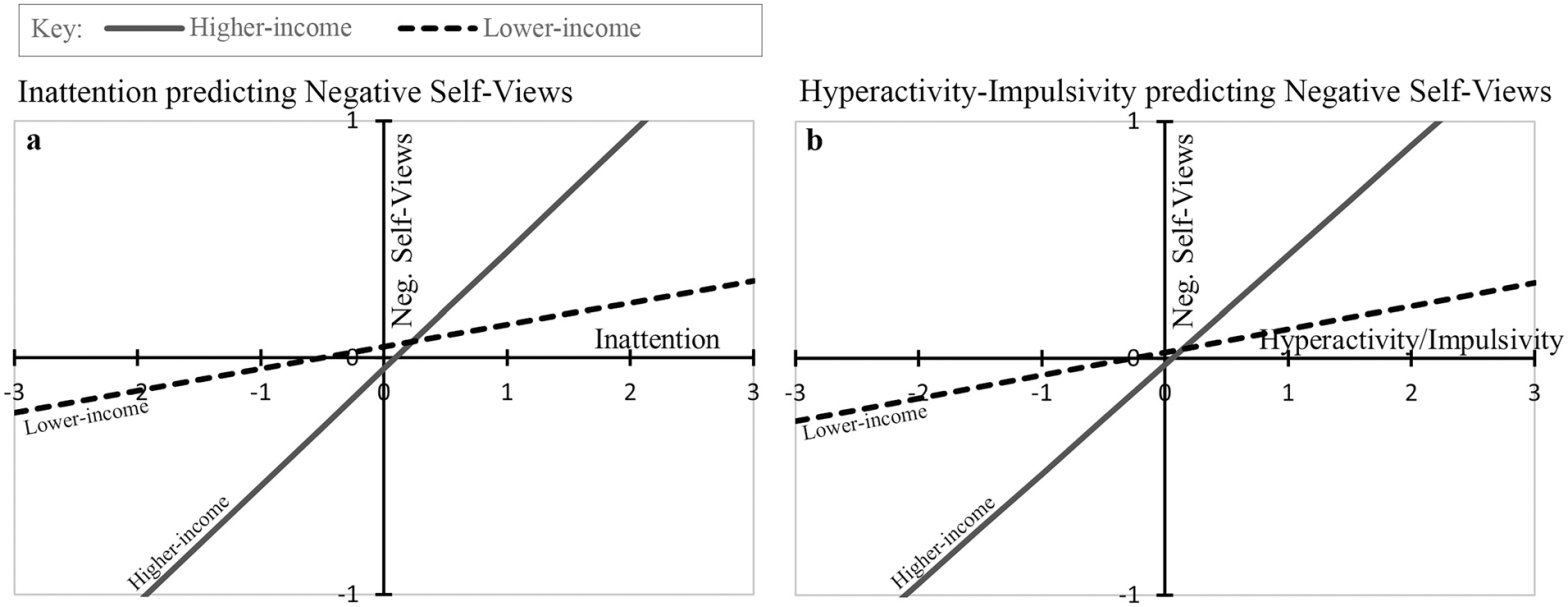
Family income as a moderator: Childhood ADHD symptom dimensions predicting negative self-views in adolescence for families with higher and lower incomes. Note. Inattention, hyperactivity/impulsivity, negative self-views, and family income were all z-scored. Higher-income families had income levels 1 SD above the mean and lower-income families 1 SD below the mean.

Results from the moderated multiple regressions predicting negative self-views are shown in Table 3. In the first analysis, IA had the expected effect, *β* = 0.29, and family income was not a significant predictor, *β* = -0.05. Most important, the interaction was significant, *β* = 0.20, indicating that IA had a stronger link to negative self-views for higher-income than for lower-income families. This pattern is illustrated in Fig. 1, panel a, revealing that the regression line was steeper for higher-income families.

**Table 3:** Family Income as a Moderator of the Negative Effects of Childhood ADHD Symptoms on Adolescents’ Self-Views: Standardized Beta Weights Predicting Negative Self-Views from ADHD Symptoms, Family Income, and their Interaction

ADHD symptom dimension		Family income	Interaction: symptom x income
Inattention	0.29 (*p* < 0.001)	-0.05 (*ns*)	0.20 (*p* = 0.003)
Hyperactivity- Impulsivity	0.28 (*p* < 0.001)	-0.03 (*ns*)	0.18 (*p* = 0.008)

*N* = 200. Family income was coded as follows: 1 = < $10,000, 2 = $10–20,000, 3 = $20–30,000, 4 = $30–40,000, 5 = $40–50,000, 6 = $50–60,000, 7 = $60–70,000, 8 = $70–75,000, 9 = > $75,000. Inattention, hyperactivity-impulsivity, negative self-views, and family income were all z-scored. Negative self-views were indexed as the mean of the reverse-coded Conscientiousness z-score, reverse-coded Agreeableness z-score, and Neuroticism z-score; that is, participants with high scores on negative self-views described themselves as lazy (low Conscientiousness), rude to others (low Agreeableness), and moody (high Neuroticism).

*ns* not significant

The same pattern was observed in the second analysis: HI had the expected effect on negative self-views, *β* = 0.28, family income was not a significant predictor, *β* = -0.03, and the interaction was significant, *β* = 0.18. This pattern is illustrated in Fig. 1, panel b, showing that HI also had a stronger effect for higher-income than for lower-income families. For those readers who may desire more granularity, in supplemental materials (see Table S3, Figure S1), we display regression betas and graphically display interactions of income with Conscientiousness, Agreeableness, and Neuroticism separately.

### Robustness Checks

In addition, we conducted robustness tests, adjusting for the effects of age and race/ethnicity, for the regression analyses in both Tables 2 and 3. As in previous research, age correlated with the Big Five dimensions at Wave 2, but these correlations were all small, none of them reaching 0.20, which is consistent with the findings in a very large sample of adolescents (Soto et al., 2011). Nonetheless, we conducted the regressions in Tables 2 and 3, covarying age. The regression estimates were essentially unchanged, and all of the effects remained significant.

In the second robustness test, we statistically adjusted for racial/ethnic differences, using dummy-coded variables for our three largest groups: Caucasian (versus not), African-American (versus not), and Latinx (versus not). None of these was correlated with the personality variables of interest, and the regression results remained significant after covarying race. As highlighted by a reviewer, this is an important analysis because income is potentially confounded with racial/ethnic differences.

## Discussion

Below, we discuss key findings before proceeding to wider implications.

### Childhood ADHD, IA, and HI Longitudinally Predicted Adolescent Personality Dimensions

First, longitudinal correlations were in the expected directions between childhood ADHD (and its core symptom dimensions, IA and HI) and the adolescent personality dimensions of Conscientiousness (negative), Agreeableness (negative), and Neuroticism (positive), with no significant correlations with adolescent Openness to Experience or Extraversion. The directions of these correlations are consistent with the well-established cross-sectional literature on the topic (Gomez & Corr, 2014).

Whereas we found robust longitudinal correlations between childhood ADHD and adolescent personality, Miller et al. (2008) found only *concurrent* but not longitudinal correlations between late adolescent personality and ADHD severity. One possible explanation includes the age of participants at the time of data collection for personality traits: In the present study, participants were mostly in early-to-mid-adolescence, *M* = 14.2 years, when their personalities were still developing (Soto et al., 2011). In contrast, in Miller et al.’s (2008) study, participants were in late adolescence to emerging adulthood, *M* = 18.4 years, when their personalities may have reached greater stability. Another potential explanation includes differences in sample composition: The present study focused on understudied females with a mix of ADHD presentations, whereas Miller et al. (2008) studied a sample that was mostly (88%) male and all diagnosed with the ADHD-combined type. Finally, the studies differ in the way ADHD was diagnosed and personality was measured. To our knowledge, ours is the second study to investigate these correlations longitudinally, and we did so in an all-female sample. It is possible that the experience of having childhood ADHD symptoms could reveal a more negative predictive effect on adolescent personality for girls than for boys; it is also conceivable that these predictions from childhood ADHD to adolescent personality do not extend into late adolescence/emerging adulthood. Clearly, additional research is needed.

### Longitudinal IA-Conscientiousness and HI-Agreeableness Predictions Were Robust to Covarying ADHD Diagnosis

Next, when pitting each ADHD symptom dimension against the ADHD diagnosis, we found that, independent of ADHD, IA negatively predicted Conscientiousness and HI negatively predicted Agreeableness, but neither IA nor HI predicted Neuroticism. The independent prediction of Conscientiousness by IA (even with item overlap removed) suggests strongly that these two measures are tapping similar domains of behavior and personality. Similarly, literature indicates that the HI dimension is more externalizing in nature than is IA (e.g., Connor et al., 2010; Eiraldi et al., 1997; Taylor et al., 1996), which may explain its negative association with Agreeableness independent of IA and the ADHD syndrome.

### Longitudinal Predictions Were Moderated by Family Income

We analyzed whether consideration of family income would accentuate longitudinal relations between childhood ADHD (and its symptom domains) and adolescent negative self-views, an aggregate of self-reported personality dimensions. Higher annual family-of-origin income was, in fact, a significant moderator in our analyses including both IA and HI as predictors: Key linkages between childhood ADHD symptoms and adolescent personality dimensions were stronger for participants from higher-income families than those from lower-income families (see Fig. 1). As one potential explanation, Luthar (2003) and Korous et al. (2023) found linkages between a “culture of affluence” and poorer youth psychological wellbeing in several key areas (e.g., higher rates of depression, anxiety, and substance use disorders) than in the general population. Core mechanisms may include high familial pressure to achieve academic/extracurricular success, a tendency to derive self-worth from one’s achievements, poor-quality parent–child relationships, and youth perceptions that parents value them more for achievements than for who they are as people (see Luthar, 2003). Although our findings are clearly exploratory, it is possible that relatively higher family socioeconomic status may enhance predictive associations between childhood ADHD and adolescent personality maladjustment.

### Strengths and Limitations

As noted above, prior work in this area has been mostly cross-sectional or retrospective—with the notable exception of Miller et al. (2008), who prospectively examined our variables of interest in a predominantly male sample. Our research builds on this work in novel and important ways, extending these findings to females and separating predictions related to ADHD symptom dimensions (IA, HI) from those related to the overall ADHD syndrome. The present findings reveal prospective relations between observed childhood ADHD (as measured by adult informants) and self-reported adolescent personality dimensions in an all-female sample. Other strengths include combining multiple observer-reports to establish presence of childhood ADHD symptoms, using different data sources at different time points to assess ADHD and personality (thus minimizing shared method variance), and adopting a conservative approach to address potential artifactual overlap between informant observer ADHD ratings and personality self-reports by deleting items that were essentially “copies” of each other.

One limitation is that childhood personality measures were not collected, as there was limited literature on the validity of self-reported personality in children at the time of study inception in the late 1990s (see Measelle et al., 2005, for one of the earliest studies examining validity of personality self-report in children). Thus, we cannot measure change in personality from childhood to adolescence or test the directionality of the relations between ADHD symptoms and personality across this developmental span. Note that our sampling frame also precluded measures of temperament during infant/toddler/preschool years. Future work would ideally take baseline temperament/personality into account. Second, there is considerable overlap between the constructs of IA and Conscientiousness. In fact, three of the nine IA symptoms (as described by the *DSM-IV*) overlap directly with Big Five Conscientiousness items, and several others are conceptually similar (see Appendix A, Table S2). Thus, we were assiduous in removing any overlapping items.

A third limitation is that the age ranges for each wave of data collection are relatively wide. Even though covarying age did not alter our findings, future researchers may want to examine narrower age ranges. Fourth, when using family income as a moderator, we did not have strong a priori hypotheses. Thus, these moderator effects need to be replicated, especially in samples containing male participants. Additional limitations specific to our moderation analysis involving income include that our analyses treat our ordinal family income variable as continuous in our moderation analyses and that our income ranges had overlapping endpoints (e.g., $10,000 to $20,000 and $20,000 to $30,000), such that participants with incomes at these endpoints (e.g., $20,000) could correctly select either range.

### Theoretical Issues

To assist with interpretation of our findings, we first highlight the BFI administration itself. An adolescent completing a self-reported BFI was asked to respond to each question—“I see myself as someone who…” (for example, “is talkative”)—and then indicate her level of agreement with that statement. One way of defining personality (at least when examining self-report data) is ‘how an individual perceives herself,’ which may or may not directly correspond with patterns of thinking, feeling, and behaving as perceived by others or objectively observed. Indeed, McCrae and Costa (1999) include the self-concept as part of the personality system, which lends support to this perspective. One framing of our findings is that having ADHD in childhood may lead to negative self-perceptions in later life. Our sample of girls with childhood ADHD tended, around five years later, to perceive themselves more negatively than their typically-developing peers–specifically, as less conscientious (i.e., less planful and organized), less agreeable (i.e., less helpful, kind, and cooperative), and more neurotic (i.e., more moody, depressed, and anxious). Domain-specifically, girls high in childhood IA viewed themselves as less conscientious than those lower in childhood IA, and girls higher in childhood HI tended to subsequently view themselves as less agreeable than those with lower HI.

At a conceptual level, debate is ongoing as to whether (i) ADHD and personality separately stem from early temperament; (ii) ADHD symptoms shape personality over the course of development via neurobiological mechanisms in concert with dynamic interactions with the environment; (iii) personality shapes ADHD symptoms over time; or even whether (iv) ADHD is an “extreme personality trait” (Nigg et al., 2002). At this point, we cannot answer these questions, but the present study provides an initial piece of the puzzle, suggesting strong links between ADHD and self-reported personality spanning childhood to adolescence.

We highlight that youth with ADHD remain at far higher risk than typically developing children for a number of serious life impairments. Crucially, girls with ADHD are at substantially increased risk for self-harm (both non-suicidal self-injury and attempted suicide) compared to those without ADHD by early adulthood (see Babinski et al., 2011; Balázs et al., 2018; Fitzgerald et al., 2019; Hinshaw et al., 2012, 2022; Hinshaw, 2018). They are also at increased risk for broad-based underachievement at school and underperformance at work, as well as four-fold greater risk for unplanned pregnancy, even when ADHD symptoms have remitted (Owens & Hinshaw, 2020; Owens et al., 2017). A next step is to examine the linkages between adolescent personality traits and such important outcomes, including a test of whether adolescent personality dimensions partially or fully mediate the predictive associations from childhood ADHD to these important clinical outcomes later in life.

Indeed, it may be that negative self-perceptions emanating from childhood ADHD are a potential mechanism by which difficult adult outcomes are perpetuated, perhaps via a “scar” model (Bagby et al., 2017). Indeed, links between low self-esteem/perceived self-competence in childhood and later suicide ideation and attempts lend support to this hypothesis (Meza et al., 2021). Personality shows considerable malleability (Srivastava et al., 2003), so that if personality (or “identity”/self-concept) accounts for relations between childhood ADHD and adult dysfunction, it could potentially become an intervention target (e.g., inoculation against negative self-perceptions via psychoeducation, targeted skill building, and/or enhancement of strengths).

## Open Science Statement

As described above, these data emanate from a large, multi-wave and multi-measure longitudinal study. Thus, it is not possible to post all these data online. However, we will make the data used in this report available upon request.

The measures used here and the procedures we followed are all standard and well-known in the personality and ADHD literature, including the BFI (John & Srivastava, 1999), the SNAP-IV (Swanson, 1992), the DISC-IV (Shaffer et al., 2000), and the measurement of family income. All these measures are freely available and could thus be used to replicate the assessments and findings presented here.

The analyses presented in this paper were conducted with SPSS version 28. The syntax used can be found at this link: https://osf.io/a3knx/?view_only=3a64e9da8477480abf9e69c6be97324f

## Supplementary Information

Below is the link to the electronic supplementary material.

Supplementary file1 (DOCX 157 KB)

## Footnotes

1 Heritability estimates for Conscientiousness, Agreeableness, and Neuroticism range from 31 to 37% (Vukasović & Bratko, 2015). In contrast, heritability estimates for ADHD range from 74 to 80% for IA and HI and from 77 to 88% for the categorical ADHD diagnosis (Faraone & Larsson, 2019).

2 Correlation sizes reported in this article are reported with Funder and Ozer’s (2019) “New Guideline” thresholds in mind: *r* = 0.05, very small; *r* = 0.10, small; *r* = 0.20, medium; *r* = 0.30, large; *r* = 0.40 or greater, very large.
