# Supplementary material for: ADHD Symptoms in Childhood and Big Five Personality Traits in Adolescence: A Five-Year Longitudinal Study in Girls
Source: Res Child Adolesc Psychopathol. 2024 May 29;52(9):1369–82. doi: 10.1007/s10802-024-01204-x (PMC11420272; doi:10.1007/s10802-024-01204-x)
Supplement: Supplementary file 1 — Supplementary file1 (DOCX 157 KB) [file 10802_2024_1204_MOESM1_ESM.docx]

# Appendix

Supplemental (S) Materials

## Table S1

*Means, Standard Deviations, and Cohen’s* d *by Sample Subset*

|  | |  | FullSample | W1 Diagnostic Groups | | | W1 ADHD Subgroups | | |
| --- | --- | --- | --- | --- | --- | --- | --- | --- | --- |
|  | |  |  | Comp-arison | ADHD | *d* | Inatt- entive | Comb-ined | *d* |
| Wave 1 | | *n* | 204 | 82 | 122 |  | 40 | 82 |  |
|  | IA | *M* | 1.36 | 0.32 | 2.06 | 4.03 | 2.13 | 2.03 | 0.19 |
|  |  | *SD* | 0.96 | 0.27 | 0.51 |  | 0.52 | 0.51 |  |
|  | HI | *M* | 0.96 | 0.20 | 1.48 | 2.42 | 0.85 | 1.78 | 1.90 |
|  |  | *SD* | 0.82 | 0.22 | 0.66 |  | 0.34 | 0.55 |  |
| Wave 2 | | *n* | 204 | 82 | 122 |  | 40 | 82 |  |
|  | IA | *M* | 1.11 | 0.44 | 1.56 | 1.83 | 1.64 | 1.52 | 0.17 |
|  |  | *SD* | 0.82 | 0.44 | 0.70 |  | 0.57 | 0.76 |  |
|  | HI | *M* | 0.53 | 0.12 | 0.80 | 1.40 | 0.57 | 0.91 | 0.58 |
|  |  | *SD* | 0.59 | 0.19 | 0.61 |  | 0.38 | 0.67 |  |
|  | C | *M* | 3.50 | 3.80 | 3.30 | 0.71 | 3.23 | 3.34 | 0.15 |
|  |  | *SD* | 0.73 | 0.65 | 0.72 |  | 0.73 | 0.72 |  |
|  | A | *M* | 3.86 | 4.00 | 3.76 | 0.40 | 3.86 | 3.71 | 0.24 |
|  |  | *SD* | 0.62 | 0.52 | 0.66 |  | 0.52 | 0.71 |  |
|  | N | *M* | 2.64 | 2.48 | 2.74 | 0.39 | 2.63 | 2.80 | 0.25 |
|  |  | *SD* | 0.68 | 0.66 | 0.67 |  | 0.54 | 0.73 |  |
|  | O | *M* | 4.07 | 4.09 | 4.05 | 0.07 | 3.83 | 4.17 | 0.62 |
|  |  | *SD* | 0.54 | 0.50 | 0.56 |  | 0.66 | 0.48 |  |
|  | E | *M* | 3.71 | 3.66 | 3.74 | 0.12 | 3.59 | 3.82 | 0.35 |
|  |  | *SD* | 0.67 | 0.68 | 0.66 |  | 0.65 | 0.66 |  |

*Note*. Sample sizes reported here represent the *n*’s for the core analyses (i.e., longitudinal correlations, hierarchical regressions). As represented in Table S2, item overlap between inattention and Conscientiousness was removed. C = Conscientiousness, A = Agreeableness, N = Neuroticism, O = Openness to experience, E = Extraversion.

## Table S2

*Overlapping Items between SNAP-IV and BFI*

| SNAP-IV Inattention items (9) |  | BFI Conscientiousness items (9) |
| --- | --- | --- |
| **1. Fails to give close attention to details or makes careless mistakes in schoolwork, work or other activities** |  | 3. Does things carefully and completely ^a^ |
|  |  | **8. Can be somewhat careless** † |
|  |  | 34. Does things efficiently (quickly and correctly) ^a^ |
| 2. Has difficulty sustaining attention in tasks or play activities |  | 29. Keeps working until things are done ^a^ |
| 3. Does not seem to listen to what is being said to him or her |  |  |
| 4. Does not follow through on instructions and fails to finish schoolwork, chores, or duties in the workplace (not due to oppositional behavior or failure to understand instructions) |  | 13. Is a reliable worker ^a^ |
|  |  | 39. Makes plans and sticks to them ^a^ |
| **5. Has difficulty organizing tasks and activities** |  | **18. Tends to be disorganized** † |
| 6. Avoids, expresses reluctance about, or has difficulties engaging in tasks that require sustained mental effort (such as schoolwork or homework) |  | 24. Tends to be lazy |
| 7. Loses things necessary for tasks or activities (e.g., school assignments, pencils, books, tools, or toys) |  |  |
| **8. Is easily distracted** † |  | **45. Is easily distracted; has trouble paying attention** |
| 9. Is forgetful in daily activities |  |  |

*Note*. SNAP-IV = Swanson, Nolan, and Pelham Scale, 4^th^ edition. BFI = Big Five Inventory.

All items from the SNAP-IV Inattention scale and BFI conscientiousness scale are reproduced here and grouped by conceptual overlap. Seriously overlapping items are bolded. For each pair of seriously overlapping items across scales, one item was randomly removed from one of the two scales for all the analyses.

† = item removed from the scale.

^a^ = opposite direction of SNAP-IV item.

## Table S3

*Summary of Moderator Analyses: Standardized Beta Weights Predicting the Big Five Traits*

*in Adolescence from ADHD Symptoms in Childhood, Family Income, and their Interaction*

| Outcome: |  | |  | Interaction: | Covariate: | Δ*R*^2 a^ |
| --- | --- | --- | --- | --- | --- | --- |
| Big Five trait | ADHD symptom dimension | | Family income | symptom x income | Age (W2) |  |
| Conscientiousness | Inattention | -.36 (*p* < .001) | .04 (ns) | -.16 (*p* = .015) | -- | .16 (*p* < .001) |
| Conscientiousness | Hyperactivity/  Impulsivity | -.20 (*p* = .005) | .05 (ns) | -.14 (*p* = .044) | -- | .06 (*p* = .008) |
|  |  |  |  |  |  |  |
| Agreeableness | Inattention | -.19 (*p* = .007) | .01 (ns) | -.19 (*p* = .006) | -- | .07 (*p* = .002) |
|  |  |  |  |  |  |  |
| Agreeableness | Hyperactivity/  Impulsivity | -.27 (*p* < .001) | -.03 (ns) | -.15 (*p* = .028) | -- | .09 (*p* < .001) |
|  |  |  |  |  |  |  |
| Neuroticism | Inattention | .13 (*p* = .066) | -.06 (ns) | .11 (ns) | .11 (ns) | .06 (*p* = .025) |
|  |  |  |  |  |  |  |
| Neuroticism | Hyperactivity/ Impulsivity | .20 (*p* = .004) | -.03 (ns) | .12 (*p* = .092) | .15 (*p* = .035) | .08 (*p* = .004) |
|  |  |  |  |  |  |  |

*Note*. *N* = 200. Inattention, hyperactivity/impulsivity, conscientiousness, agreeableness, neuroticism, and family income are all z-scored. Prior to *z*-score transformation, family income was coded as follows: 1 = < $10,000, 2 = $10-20,000, 3 = $20-30,000, 4 = $30-40,000, 5 = $40-50,000, 6 = $50-60,000, 7 = $60-70,000, 8 = $70-75,000, 9 = > $75,000.

ns = not significant

^a^ *p*-values in this column refer to the significance of the ΔF-statistic.

## Figure S1

*Family income as a moderator: Childhood ADHD symptoms predicting adolescent personality

traits separately for families with higher income and lower income*

| Key: 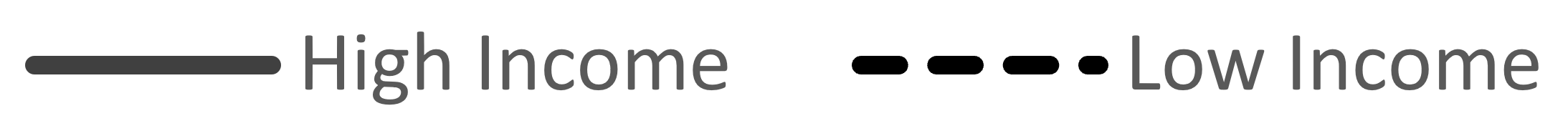High Income 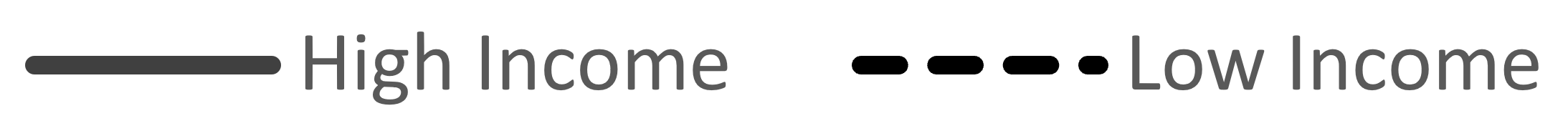Low Income | |
| --- | --- |
| IA predicting Conscientiousness | HI predicting Conscientiousness |
| 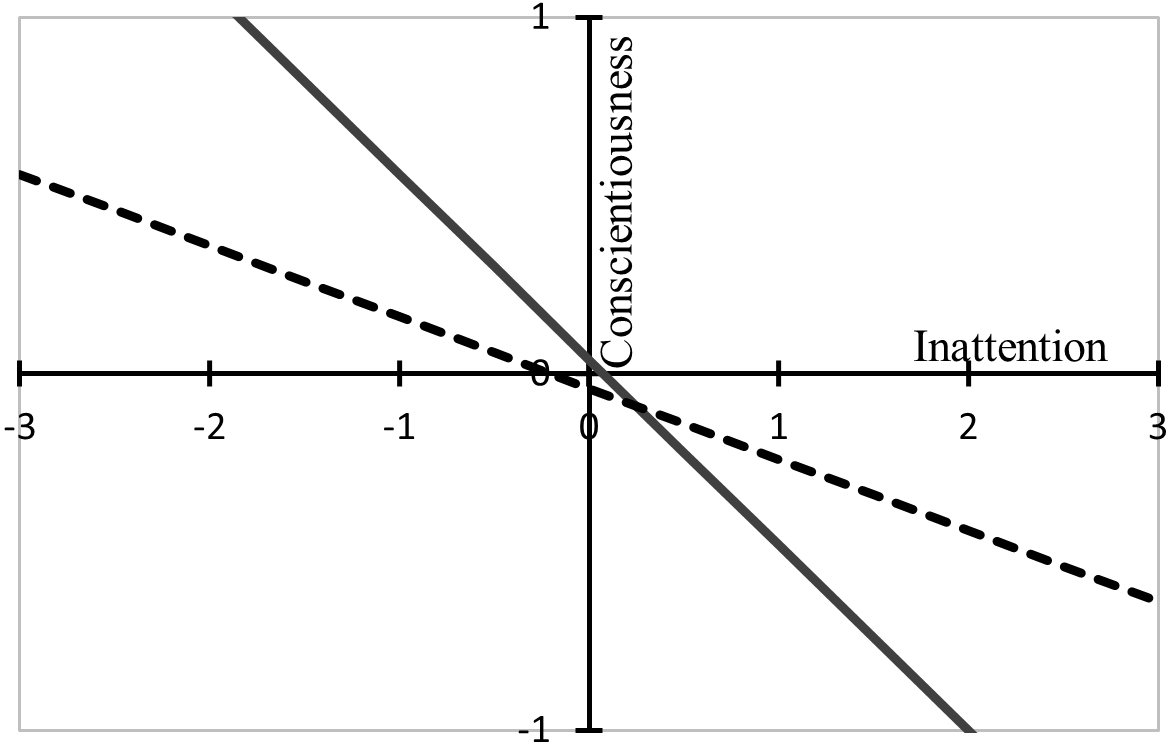  $\hat{C}_{high inc}=-.52\left( ia \right)+.04$ $\hat{C}_{low inc}=-.20\left( ia \right)-.04$  **a** | 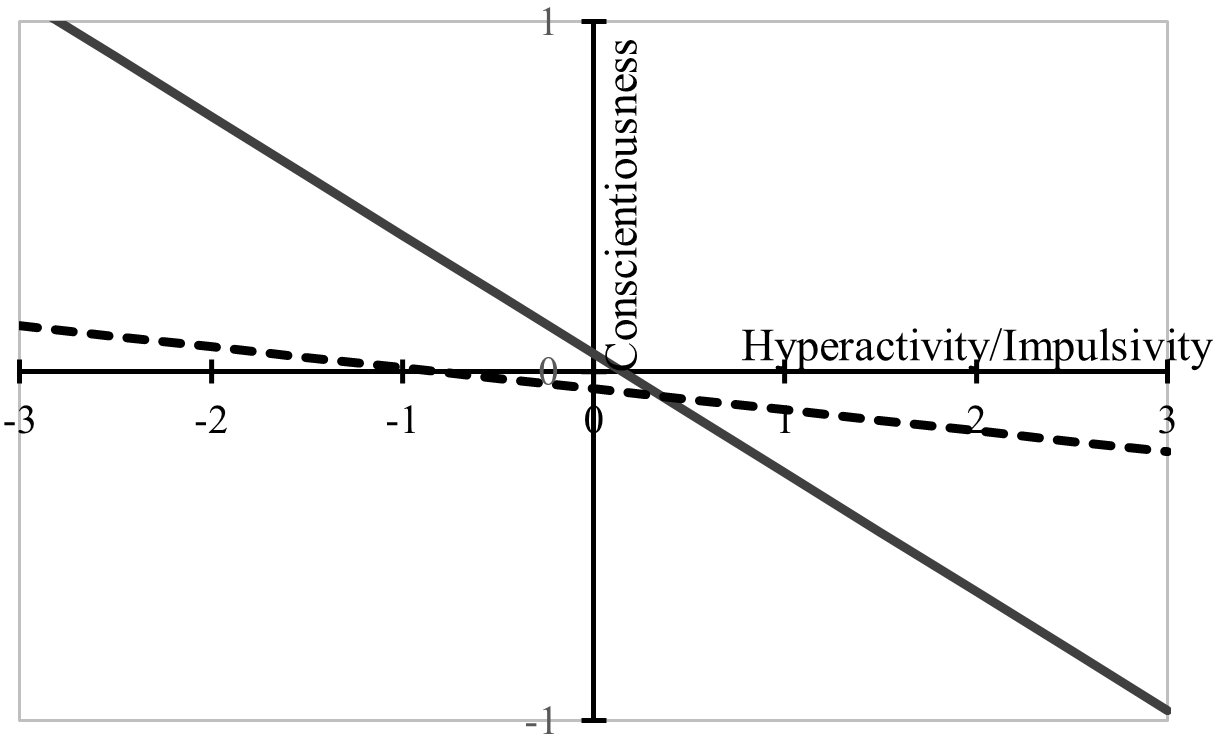  **b**  $\hat{C}_{high inc}=-.34\left( hi \right)+.05$ $\hat{C}_{low inc}=-.06\left( hi \right)-.05$ |
| IA predicting Agreeableness | HI predicting Agreeableness  **d** |
| 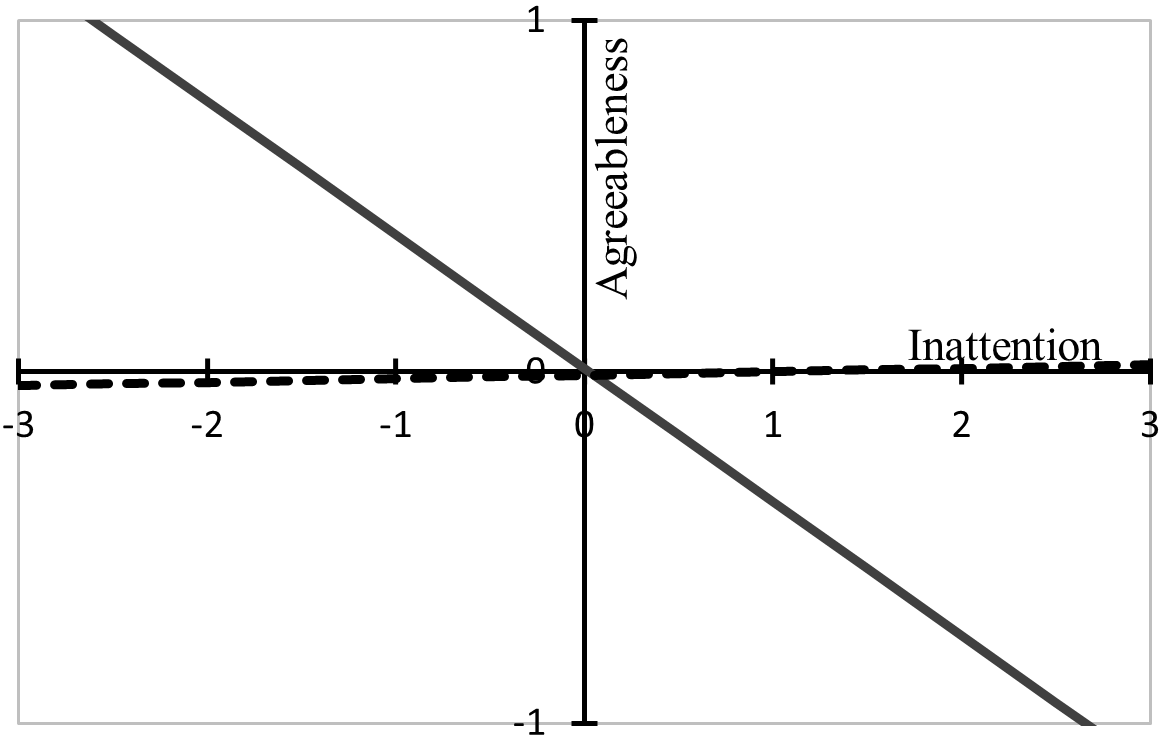  $\hat{A}_{high inc}=-.38\left( ia \right)+.01$ $\hat{A}_{low inc}=.01\left( ia \right)-.01$  **c** | 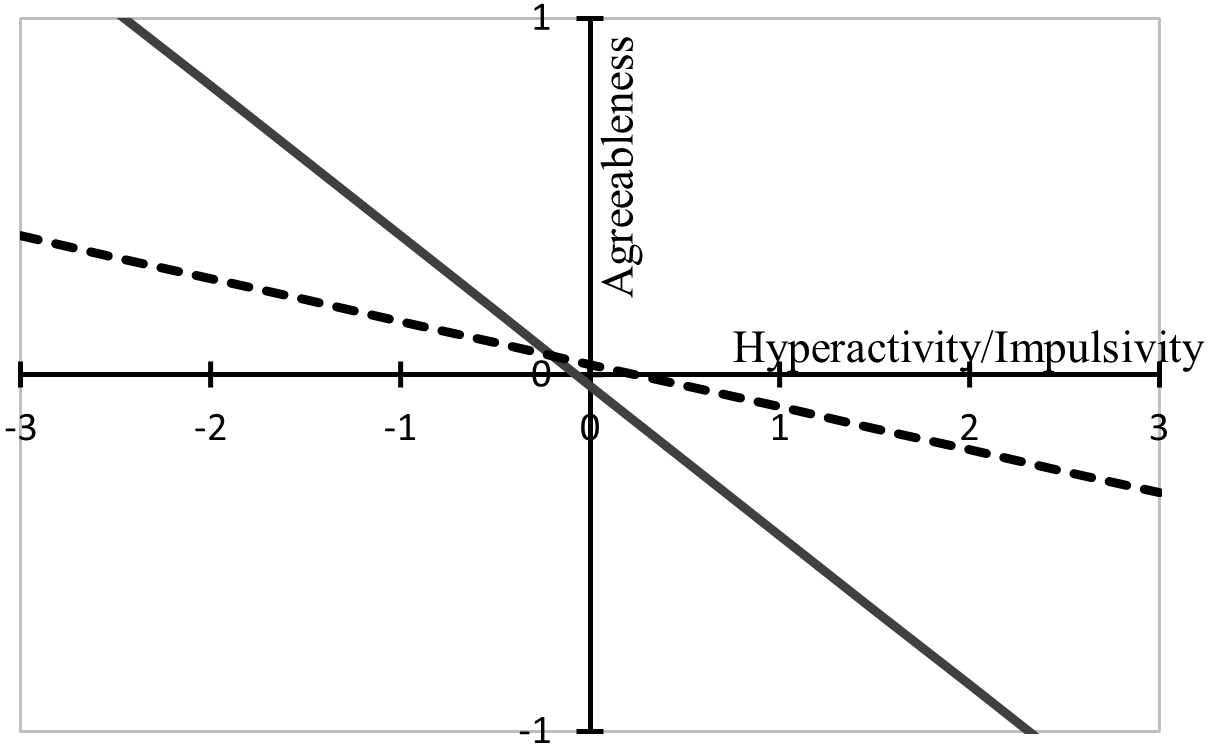  $\hat{A}_{high inc}=-.42\left( hi \right)-.03$ $\hat{A}_{low inc}=-.12\left( hi \right)+.03$ |
| IA predicting Neuroticism | HI predicting Neuroticism  **f** |
| 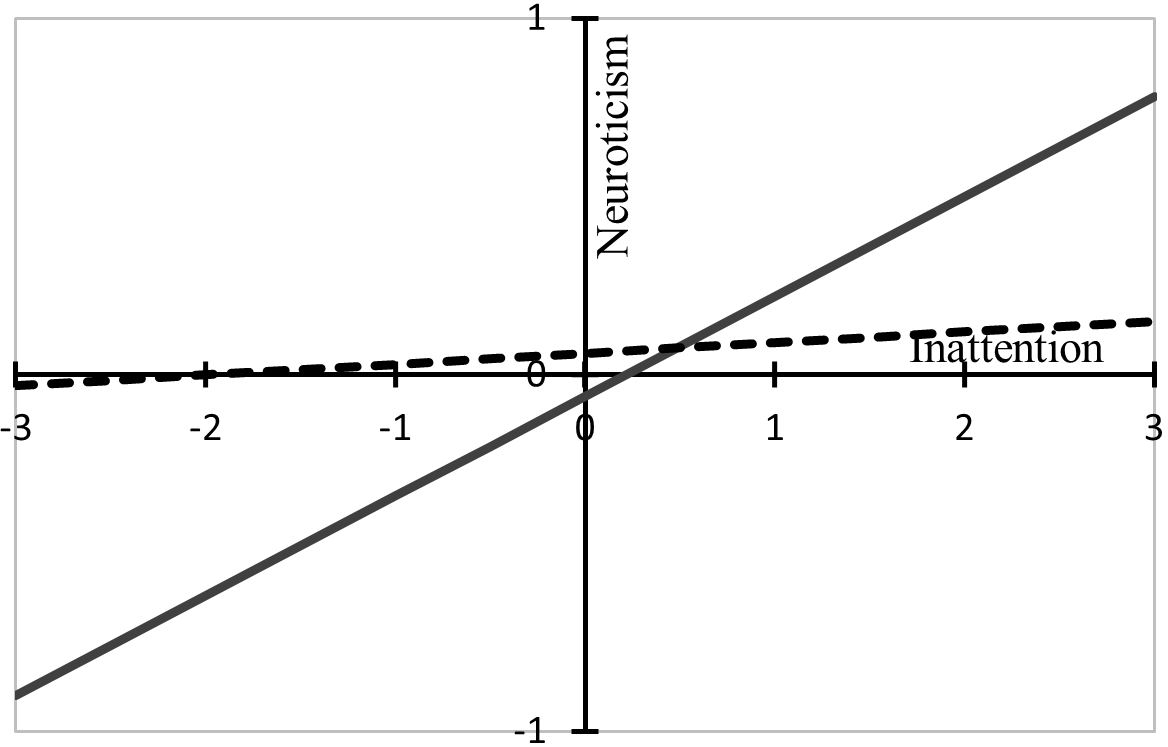  $\hat{N}_{high inc}=.28\left( ia \right)-.06$ $\hat{N}_{low inc}=.03\left( ia \right)+.06$  **e** | 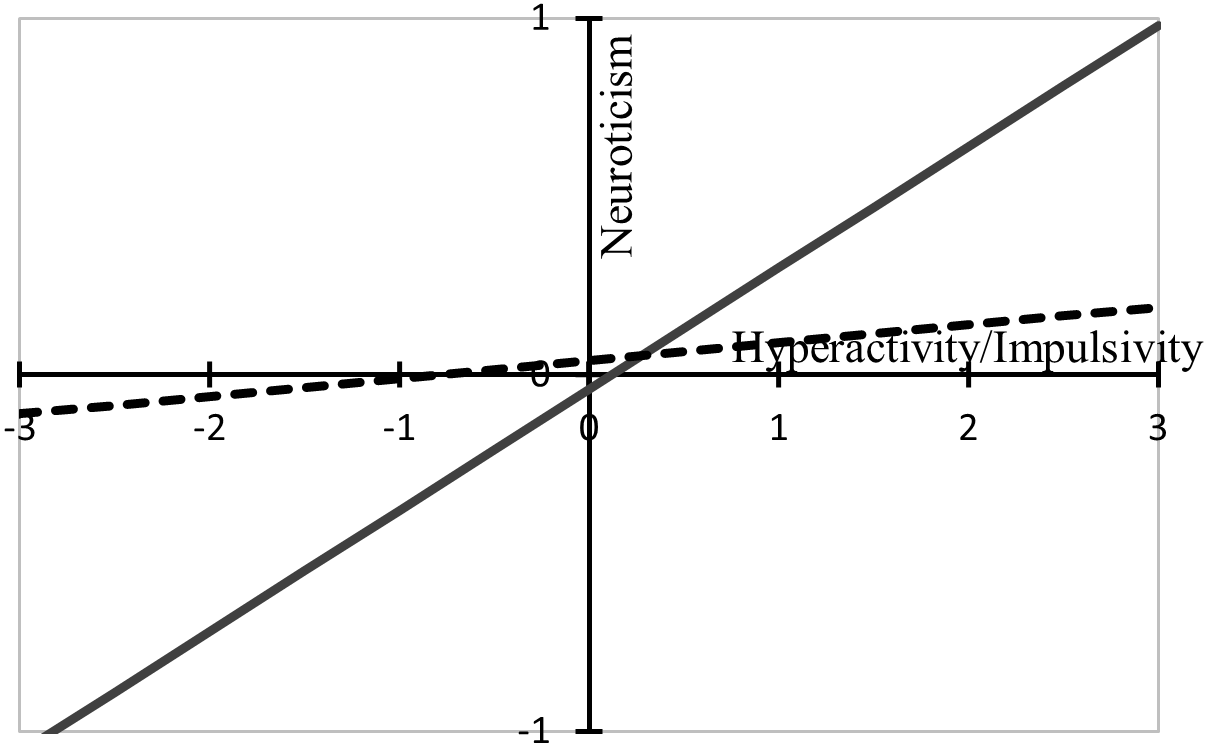  $\hat{N}_{high inc}=.34\left( hi \right)-.04$ $\hat{N}_{low inc}=.05\left( hi \right)+.04$ |
|  |  |

*Note.* Inattention (IA), hyperactivity-impulsivity (HI), Conscientiousness, Agreeableness, Neuroticism, and family income are all z-scored. Affluent families had income levels ≥ 1 SD above the mean. Low income families had income levels ≥ 1 SD below the mean.
